# Clinical simulation for the development of communication and teamwork in nursing*

**DOI:** 10.1590/1518-8345.7560.4548

**Published:** 2025-07-28

**Authors:** Aline Teixeira Silva, Laura Andrian Leal, Maria Inês Lemos Coelho Ribeiro, Monise Martins da Silva, Jeniffer Stephanie Marques Hilário, Sílvia Helena Henriques

**Affiliations:** 1 Universidade do Estado de Minas Gerais, Unidade Passos, Departamento de Enfermagem, Passos, MG, Brazil.; 2 Universidade de São Paulo, Escola de Enfermagem de Ribeirão Preto, PAHO/WHO Collaborating Centre for Nursing Research Development, Departamento de Enfermagem Geral e Especializada, Ribeirão Preto, SP, Brazil.; 3 Universidade do Estado de Minas Gerais, Unidade Passos, Departamento de Ciências Médicas, Passos, MG, Brazil.; 4 Universidade de São Paulo, Escola de Enfermagem de Ribeirão Preto, PAHO/WHO Collaborating Centre for Nursing Research Development, Ribeirão Preto, SP, Brazil.

**Keywords:** Simulation Training, Education Interprofessional, Communication, Patient Care Team, Students, Nursing, Nursing Education

## Abstract

to implement a realistic simulation scenario aimed at developing communication and teamwork skills in nursing undergraduates and analyzing its contributions during hospital internships.

exploratory study conducted with 27 final-year nursing students from a higher education institution. Data were collected in 2021 using a checklist and a validated scenario centered on communication and teamwork in the hospital setting. Data collection took place in two stages: first, during the execution of the scenario/debriefing in the institution’s skills lab, and later, after the completion of the hospital internship, through semi-structured interviews. The debriefing and interviews discussions were analyzed through inductive thematic analysis.

during the debriefing stage, the undergraduate students reflected on their participation in the scenario, the case resolution, and their experiences with communication and teamwork. In the interviews, the students expressed a desire to include additional academic disciplines in the simulated classes and highlighted the benefits of using simulation in hospital practice.

the simulation deepened the students’ understanding by fostering critical and reflective thinking on communication and teamwork during their hospital internship, thus highlighting the value of its implementation throughout their university education.

## Highlights

**(1)** Implementation of clinical simulation in students prior to hospital practice. **(2)** Improvement of communication and teamwork in nursing students. **(3)** Expanding knowledge using realistic laboratory activities. **(4)** Broadening the student’s view of the nurse’s role in the hospital environment **(5)** Discussion of feelings and case resolution favored by Debriefing.

## Introduction

Nurses, as essential healthcare professionals, play a vital role in patient care by addressing needs through their expertise and competencies^(1)^. In other words, they aim to apply their knowledge, skills, and attitudes effectively in the workplace. In the hospital context, a highly complex environment, these professionals have progressively worked to enhance their competencies, aiming to promote interprofessional collaboration^(2)^.

In this context, scholars suggest that ongoing education and training centered on interprofessional practice can foster the development of competencies that enhance collaborative practice^(3)^. It is emphasized that interprofessional competencies encompass clarity of roles and responsibilities, values, ethics, communication, teamwork, conflict resolution, leadership, and patient-centered care, involving patients, their families, and society^(4-5)^. Therefore, the study highlights two key competencies for nurses’ effective collaboration in healthcare settings: communication and teamwork. Communication is considered a crucial element in building strong interpersonal relationships between caregivers, healthcare users, and their families, while teamwork promotes the integration of various professions and specialties^(4)^.

Thus, to ensure that future nurses develop their communication and teamwork skills during clinical practice, an educational space should be created for this purpose^(6-7)^. In this context, Interprofessional Education stands out as an effective strategy for acquiring these competencies. Within universities, this approach aims to engage two or more students and/or professionals, promoting collaborative learning. The goal of this learning is to better prepare them for effective collaboration in healthcare settings^(8)^.

Moreover, in today’s world, it is widely recognized that students have a natural affinity for interactive and immersive technologies, often adopting them as native digital resources. This familiarity with technology can serve as a catalyst for education, not only preparing future healthcare professionals but also advancing interprofessional training. It also helps educational institutions integrate active methodologies, such as realistic simulation.

In this context, the use of simulation can enhance students’ competencies, allowing them to reflect on real-life scenarios within a controlled environment and thereby supporting their practical training^(9-10)^. Furthermore, simulation scenarios are most effective when designed around and based on real clinical cases, as they can replicate situations commonly encountered in the daily practice of the nursing team^(11)^.

Therefore, it is acknowledged that implementing validated clinical scenarios for healthcare training aimed at enhancing interprofessional competencies in nursing undergraduates is crucial, though such initiatives are still underdeveloped in many universities across the country. In light of this, the study poses the following guiding questions: How can a healthcare training scenario (realistic simulation) be designed to enhance interprofessional competencies, particularly communication and teamwork, in nursing education? What are the positive and/or limiting aspects of the simulated activity? How do students perceive the method used? And what contributions does this learning process provide prior to the practical internship?

Meanwhile, the study aimed to implement a realistic simulation scenario to enhance communication and teamwork skills among nursing undergraduates and evaluate its contributions to their hospital internship experience.

## Method

### Study design

Exploratory study with qualitative data approach^(12)^. The article was written following the recommendations of the Consolidated Criteria for Reporting Qualitative Studies (COREQ), as outlined on the Equator Network website^(13)^.

### Study location

The study was carried out in the Skills Lab of a public university located in the municipality of Passos, MG, Brazil. The laboratory is utilized by students of medicine, nursing, nutrition, aesthetics, and biomedical sciences. The classes and activities ministered in the laboratory prepare students for clinical practice by refining their competencies. The laboratory is equipped with projectors, workstations, beds, low-fidelity simulators, and a nursing station.

### Study period

Data collection took place between May and August 2021.

### Study participants

Sixty-two students enrolled in the Bachelor’s Degree in Nursing were invited to participate, 22 in the morning shift and 40 in the evening shift. Students enrolled in the tenth period of the Bachelor’s Degree in Nursing, in the Supervised Curricular Internship II discipline, were selected. The reasons for refusal to participate in the study (n=35) were, in their vast majority, professionals (nursing technicians) and these individuals were, during the data collection period, working in health services during the COVID-19 pandemic, with altered work shifts, limiting their involvement in the research. Thus, 27 nursing undergraduates participated in the study.

### Study variables

The demographic variables chosen for the study were sex, age group, marital status, affiliation, nationality and profession, and those related to communication and teamwork were based on the guidelines of the International Nursing Association Clinical Simulation & Learning (INACSL): Simulation-Enhanced Interprofessional Education (Sim-IPE)^(14)^.

### Instruments used for data collection

A checklist and a validated clinical scenario were used, focusing on the guidelines from the International Nursing Association Clinical Simulation & Learning (INACSL): Simulation-Enhanced Interprofessional Education (Sim-IPE)^(14)^.

The checklist detailing the actions of both the facilitator and the participating students included 28 items divided into 3 domains: briefing (7 items), scenario in action (14 items), and debriefing (7 items).

The clinical scenario, titled “Communication and Teamwork in the Hospital Environment”, was designed to simulate nursing care for a patient admitted to a Medical Clinic unit, with an emphasis on developing assertive communication and teamwork in managing an incident (allergic reaction). The scenario also outlined the essential components for its execution, including: the duration of each simulation stage (Briefing, Scenario in Action, and Debriefing); the clinical case definition; the patient’s details and conditions and the tasks students were expected to perform. It also specified the care setting, the characters/actors (doctor, patient, companion, and night shift nurse), as well as the materials and equipment involved.

For the interviews, a pre-designed script was used after the scenario was implemented, containing four questions: 1) What was your experience participating in clinical simulations during your undergraduate studies? 2) Do you think that simulations are important for the development of interprofessional competencies? In what way? 3) Would you say that the development of the simulated scenario “Communication and Teamwork in the Hospital Environment” brought any contribution to your performance during the practical internship? If so, what were they? 4) In which situations and/or settings were you able to develop these competencies? What were your strengths and challenges?

### Data collection

Data were collected from the nursing students in two stages. First, a theoretical activity on the topic was conducted, followed by clinical simulation, with data gathered during the debriefing stage. Subsequently, after the completion of the Supervised Internship II course, individual semi-structured interviews were conducted to assess the impact of the scenario on the students’ hospital practice. The theoretical class lasted up to twenty minutes, while the simulation was divided as follows: briefing – five minutes, scenario in action – ten minutes, and debriefing – twenty minutes, for a total duration of thirty-five minutes.

The main researcher, a nurse and nursing educator with expertise in the field, served as the facilitator for this scenario. To carry out the study, she took part in postgraduate courses on active methodologies and realistic simulation and also served as a member of the evaluation board for simulated scenarios. To further enhance the reliability of the data, a research assistant with a master’s degree in health sciences and experience as an intensive care nurse at the municipal general hospital was invited to participate in the study. It is important to note that the assistant had no affiliation with the institution and followed all ethical guidelines: he invited the students to participate in the research; observed all simulation activities, and finally, took notes and made observations on the students’ discussions during the *debriefing* stage, supporting the main researcher.

In this way, to ensure the ethical integrity of the research, the following measures were implemented: the presence of a research assistant; anonymity and confidentiality of the participants; independent informed consent; clear distinction between researcher and evaluator; and the opportunity for participants to act and express their opinions in neutral settings.

The students were invited to participate in the study through electronic messages, such as those sent via WhatsApp. Once confirmed, they received information about the dates and times of the simulation at the laboratory of the previously mentioned institution. Groups of no more than six undergraduate students were formed to take part in the simulated activity. The simulation was carried out six times, providing various options for student participation. Notably, the students who did not participate in the study (n=35) were primarily professionals (nursing technicians) who, during that period, were working in healthcare services amid the COVID-19 pandemic, with irregular work shifts that limited their ability to engage in the research.

At the start of the clinical simulation activity, that is, during the Briefing stage, the initial instructions were given; first, after inviting and selecting the students-actors, the environment where the scenario would take place was introduced, along with the objectives and the clinical case. Following that, the second stage, Scenario in Action, took place, where a situation was presented for students to develop their communication and teamwork skills in a context as close to real-life scenarios as possible.

Regarding the execution of the scenario, two students were selected to play roles: one as a nursing technician and the other as a nurse. The other participants remained as observers. After introducing the materials and equipment available in the scenario, the facilitator shared the case with all participants, both role-players and observers, and then exited the scene. At this point, the trained actors/role-players (night nurse, doctor, companion, and patient) began performing their roles.

At the end of this phase, the students participated in the Debriefing. The researcher/facilitator invited the students to sit in a semicircle and guided them through a reflective discussion on several points: the purpose of the simulation; the emotions experienced during the exercise; the positive aspects of their performance; areas for improvement, alternative approaches that could have been taken while ensuring that criticism remained constructive; and the integration of theory with practice, with a focus on communication and teamwork. Everyone involved was given an equal opportunity to share their viewpoints.

Following the academic activities, the students proceeded to the hospital-based internship. After this period, they were invited to participate in the final phase of the research, the semi-structured interview. The interview was scheduled and conducted individually at the educational institution, in a private setting, with an approximate duration of 20 minutes. Each participant was interviewed only once. All 27 students participated in the interviews, and data saturation was not reached.

### Data treatment and analysis

All the material from the Debriefing stage and the individual interviews was audio-recorded and subsequently transcribed. The transcriptions were performed manually by the researchers in charge. No software was used for data interpretation.

The data were analyzed through inductive thematic analysis. The thematic analysis was divided into six phases: 1. Familiarization with the data; 2. Code generation; 3. Theme identification; 4. Theme review; 5. Naming of themes; and 6. Drafting of the final report^(15)^.

### Ethical aspects

The research was conducted in accordance with Resolution No. 466/2012, and the participants signed the Free and Informed Consent Form (Portuguese acronym: TCLE). To protect the identities of those involved during the debriefing stage and interviews, participants were assigned identifiers using a combination of letters and numbers, such as: N1, N2, N3, and so on. The research was approved by the Research Ethics Committee of the Proposing Institution under protocol CAAE 23555819.9.0000.5393 and by the Co-participating Institution under protocol CAAE 23555819.9.3001.5112.

It is important to note that the data collected will be stored for five years, with access granted exclusively to the researchers in charge and the Research Ethics Committee (Portuguese Acronym: CEP) of the Proposing and Co-participating Institutions, upon request. After this period, all the aforementioned records will be permanently deleted to ensure that the participants’ identities remain confidential.

## Results

### Characterization of the participants

Table 1 presents the participants’ characteristics in relation to the following variables: gender, age group, marital status, kinship, place of birth, and profession.

**Table 1 -:** Sociodemographic profile of the nursing students participating in the study (n = 27). Passos, MG, Brazil, 2024

**Variables**	**Characterization**	**n**	**(%)**
Gender	Female	24	89%
	Male	3	11%
Age group	21-30 years old	22	82%
	31-40 years old	3	11%
	41-50 years old	2	7%
Marital status	Single	19	70%
	Married	7	26%
	Divorced	1	4%
Number of children	0 children	19	70%
	1 child	4	15%
	2 children	3	11%
	3 children	1	4%
Place of birth	Passos	14	52%
	Other cities	13	48%
Profession	Unemployed	14	52%
	Nursing Technician	10	37%
	Others	3	11%

Of the 27 (100%) undergraduate nursing students, the majority were female, and most were between the ages of 21 and 30. Regarding marital status, 19 (70%) were single and without children. Thirteen (48%) participants lived in other cities, and 11 (79%) of them completed their non-mandatory internships (as scholarship holders) at different health institutions in the municipality. It was also observed that ten participants were nursing technicians working at *Santa Casa de Passos*. Of these, four had been working at the institution for 1 to 5 years, three for 6 to 10 years, and three others for over 11 years. Finally, three participants reported having additional professions, such as receptionist and manicurist.

The results allowed us to identify two major thematic categories: simulated scenario for the development of communication and teamwork in nursing undergraduates and contributions of simulation to clinical practice.

### Simulated scenario for the development of communication and teamwork in nursing undergraduates

#### Regarding the simulated experience

During the execution of the simulation, the nurse on the night shift conducted a handover at the nursing station to the students-actors (nurse and nursing technician): an elderly hypertensive patient, experiencing confusion, who had been hospitalized for less than two hours with pneumonia; she had a fever spike of 37.9°C and was administered 40 drops of dipyrone orally. The patient’s daughter had just arrived with the mother’s ongoing medication and pressed the call bell. The students, upon noticing the call bell was activated, went to check the patient. At that moment, the patient’s daughter asked the students about the erythema and local itching that the patient was experiencing. In the prescription room, the doctor in charge was reviewing the patients’ records to prepare for the medical rounds.

In this context, the students were expected to draw on the knowledge gained from both their previous coursework and the theoretical module provided prior to the scenario. They were tasked with assessing the situation; delivering the necessary and appropriate nursing care based on their evaluation; ensuring clear and effective communication with the patient, their family, and the healthcare team; and contributing to the resolution of the issue presented by the patient. They were expected to apply their knowledge, skills, and attitudes to manage a potential situation that could arise in a hospital environment.

#### Debriefing

After the scenario execution, the students were invited to participate in the Debriefing stage. They had the chance to reflect on the situations they encountered during the simulation, share their emotions, evaluate the strengths of their performance, and consider what they would do differently during simulation. Thus, by applying an inductive thematic analysis in which codes and themes were derived from the students’ own narratives, it became possible to uncover new perceptions, feelings, types of knowledge, and areas for improvement during the simulation.

*We showed up, and I didn’t think there would be a situation like this to act in. At that moment, I felt a bit nervous, not knowing what to do.* (N20)

*I was a bit hesitant at first, thinking it was going to be something complicated, but it wasn’t.* (N1)

At the same time, the students expressed a sense of satisfaction in being part of the simulated experience:

*I loved it! It’s incredibly valuable to learn this here and then have the opportunity to apply it in practice. We might feel a bit nervous, but when we get to the hospital, we’ll remember what happened here—‘now I do this, this, and this’, just like I learned in class.* (N13)

In the meantime, the participants discussed how the scenario closely resembled real-life practice, thus helping them acquire knowledge.

*It was good because it brings us closer to reality, to practice. Although we’re not in the hospital setting, we’re held accountable as if we were, which makes us pay closer attention to the details, bringing the experience much closer to reality.* (N26)

*In the simulation, in addition to its closeness to reality, I find that it encourages us to think more creatively. We can engage in discussions with others and notice if their thoughts or actions differ from what we expected or what we’ve learned in textbooks.* (N24)

Based on this context, the participants had the opportunity to reflect on the positive aspects experienced in the scenario, such as problem-solving, patient reception, communication, and teamwork, as outlined below:

*I felt that my performance was appropriate for the situation; we identified the problem as soon as we entered the room, and we requested a medical evaluation. So, for that specific situation, it was the right course of action to take.* (N10)

*In terms of providing a good reception for the patient and their companion, I believe we handled that very well.* (N1)

*I also felt that there was effective communication with the doctor and the professionals in charge at that moment.* (N26)

*I believe we worked well as a team, as both of us were well integrated with the doctor.* (N2)

However, the students also highlighted negative or limiting aspects that hindered patient care. They were encouraged to reflect on whether they would approach the situation differently and how they could improve their actions. The statements highlighted aspects such as conducting a more thorough investigation of the patient’s complaint, performing a complete physical examination, ensuring proper identification, and improving their introduction to the patient.

*They should have uncovered the patient to assess the itching on her body... they asked if she was feeling anything else, but they didn’t examine her.* (N23)

*They should have included the patient’s allergy information in her identification from the beginning; it’s important to always highlight the patient’s allergies!* (N12)

*They should have introduced themselves as well; the first thing to do when entering a room is to say, “I’m [name].”* (N12)

These points were thoroughly discussed during the *Debriefing* stage, connecting theory with the practical experience gained during the simulation.

### Contributions of simulation to clinical practice

In the final stage of the research, following the students’ immersion in practical fieldwork, all 27 students were invited to take part in individual interviews conducted by the main researcher. The students acknowledged that their participation in the simulated scenario, before their hospital internships, provided them with a solid foundation for their actions during clinical practice. As a result, new codes and themes emerged from the interviews.

The students shared that they were able to communicate more effectively and collaborate better as a team. As a result, they suggested incorporating more simulations into the academic program, as reflected in the statements below.

*First, I believe there should be more simulations, as I think moving students from theory to lab simulations would help them correct their mistakes. Then, if they were subsequently exposed to real practice, I believe we would have much more qualified nurses.* (N11)

*I think professors should conduct more lab simulations; this would greatly benefit the students, make the learning process much more engaging, and help them learn more, breaking down barriers.* (N5)

Next, the students spoke about the benefits of the scenario during their immersion in the internship experience.

*I began paying more attention during the internship to the points of failure and areas worked on during the simulation, which made it easier for me to learn more about body language, speak more calmly, make eye contact, and check if the person truly understood what I was trying to say. So, I improved in these aspects.* (N2)

*One day during the internship, my colleagues forgot to introduce themselves, and that reminded me a lot of the simulation. I paid attention to that.* (N26)

*I applied [what I learned] during dialysis, which improved my communication and handover, because sometimes some things get overlooked. Now I can pay more attention to the companions of the patients, it got much easier.* (N5)

*The simulation helped me a lot because, before, we didn’t work together; it was individual work. So, after this internship, we began helping each other more, and communication improved significantly.* (N14)

Finally, the students emphasized how the experience contributed to their learning as future professionals entering the workforce.

*It will help me a lot because it gives you a different perspective on teamwork, communication, and how to interact with the medical team. So, it ends up helping us in many areas, such as taking the lead in situations, leading with confidence, and conveying that confidence to the team.* (N18)

*I believe it will help me a lot because teamwork and communication are essential in nursing; they are crucial for effectively approaching the patient. Without communication, it’s impossible, there’s no way around it! I believe it will help me feel more confident in my role as a nurse and in performing my duties.* (N23)

## Discussion

This study implemented a simulation-based experience with nursing students to enhance their communication and teamwork skills. The results revealed two main thematic categories: the simulated scenario for developing communication and teamwork in nursing students, and the contributions of simulation to clinical practice. The findings emphasized that the use of realistic simulation with nursing students is crucial for developing and refining competencies, communication, and teamwork—essential elements for professional practice and effective collaboration in the hospital setting.

It was evident that the students were able to develop their skills from the first contact with the simulation environment: they communicated and collaborated effectively with the night shift nurse, the patient and their companion, and with the doctor, while identifying the patient’s allergic reaction that required immediate attention. Nursing requires effective interaction between the team and the patient, and the simulation setting offers a controlled and safe environment where students can engage in real-life scenarios, develop conflict management skills, enhance communication assertiveness, and foster interprofessional collaboration^(16)^.

During the debriefing phase, the students’ statements revealed emotions, feelings, and perceptions of their learning experience during the simulation. This can contribute to discussions for future simulations and/or research projects while also providing valuable insights for educators to enhance or replicate such activity. Scholars assert that clinical simulation has become one of the most widely adopted methods in nursing education, as it not only helps students build knowledge and confidence in their actions but also deepens theoretical understanding through practical, hands-on scenarios^(16)^.

Although simulated activities contribute to the enhancement of skills, attitudes, and knowledge, it is recognized that they can also evoke feelings of fear, anxiety, and uncertainty^(17)^, as some level of anxiety was observed among the students in this study. Therefore, it is crucial for students to become familiar with the purpose of simulation, its objectives, the simulated environment, and the available technologies. At this stage, it is the professor’s responsibility to guide students, creating an environment that encourages the sharing of experiences and the construction of new knowledge^(18)^.

At the same time, aligning simulation with real-life practice generates strong receptiveness from students toward this methodology^(19)^, as it allows them to be immersed in situations that closely resemble reality, where they can make mistakes, repeat processes, and discuss patient care in a safe environment, leading to a more solid education by integrating theory and practice^(20)^. Thus, throughout the simulation, the process of “learning about, with, and from each other” can foster collaborative practice and promote effective care, aligned with the principles of Interprofessional Education^(14)^.

At the same time, the contextual units also revealed statements in which the students reflected on the actions they could have approached differently. They highlighted the absence of some key actions, which they identified as limitations in their participation in the scenario, such as: investigating the patient’s complaint, performing a thorough physical examination, ensuring accurate identification, and providing a proper introduction to the patient.

It is well known that a student’s development includes aspects that can be improved, which helps identify potential gaps in their learning. This emphasizes the importance of simulation^(21)^, along with the inclusion of various situations and approaches, especially in the early years of the undergraduate program, as it can encourage students to engage more effectively in discussions about the actions to be taken with the patient, their family, and the professional team. Furthermore, feedback from the instructor is crucial, as it enables students to reflect on their practices and professional behaviors^(22)^. Thus, the use of simulation made key contributions to the students’ learning process by fostering critical and reflective thinking, broadening their awareness, and encouraging shared responsibility in developing essential competencies for professional practice, such as communication and teamwork.

Therefore, by having the opportunity to experience simulation before their practical internship, undergraduate students are able to enhance their knowledge and skills, reflect on their professional role, and learn to work collaboratively, significantly contributing to the development of their professional identity^(23)^. At the same time, studies^(24-25)^ show that students who participated in simulated activities absorbed more knowledge than those who did not. This underscores the importance of regularly conducting training sessions/qualification courses to ensure long-term knowledge retention. To achieve this, a well-trained teaching staff is essential.

Researchers note that logistics and funding are the primary obstacles to adopting simulated activities in educational institutions^(26)^. Others argue that introducing clinical simulation poses a challenge for educators, as it requires time and effort to train the teaching staff, along with preparation, the definition of learning objectives, and the actual implementation of the simulation^(27)^.

However, simulated scenarios are inherently tied to the creativity and commitment of the educator, both in their design and execution, serving as a valuable teaching tool when grounded in theoretical principles^(28)^. Therefore, it is essential to incorporate discussions on the pedagogical and political frameworks of courses focused on Interprofessional Education, while also integrating realistic simulation, to effectively prepare educators and future nursing professionals for collaborative practice in healthcare settings.

The scenario presented in this study illustrates a case/event from the hospital setting, which may limit its applicability in other areas, such as public health, even though communication and teamwork are key elements of any professional environment.

This study contributes to the advancement of scientific knowledge by providing nursing undergraduate programs with the opportunity to explore and develop students’ communication and teamwork skills through a validated scenario. In this way, students can expand their knowledge by participating in a realistic activity conducted in a laboratory setting. Furthermore, simulation can help students develop a more comprehensive understanding of the nurse’s role in the hospital setting, guiding these future professionals toward safe and collaborative practice.

## Conclusion

With the goal of educating and developing/enhancing communication and teamwork skills in nursing undergraduates for interprofessional and collaborative practice, a simulated scenario was implemented involving a patient hospitalized in a medical ward, allowing students to apply these skills in response to an unexpected event. Therefore, the simulation provided students with a deeper understanding by fostering critical and reflective analysis of communication and teamwork during their hospital internship, highlighting the importance of integrating these practices into their university education.
