# Supplementary material for: Clinical simulation for the development of communication and teamwork in nursing
Source: Rev Lat Am Enfermagem. 2025 Jul 28;33:e4548. doi: 10.1590/1518-8345.7560.4548 (PMC12306934; doi:10.1590/1518-8345.7560.4548)
Supplement: Translation

# Translation: Simulação clínica para o desenvolvimento da comunicação e trabalho em equipe na enfermagem*

**DOI:** 10.1590/1518-8345.7560.4549

**Authors:** Aline Teixeira Silva, Laura Andrian Leal, Maria Inês Lemos Coelho Ribeiro, Monise Martins da Silva, Jeniffer Stephanie Marques Hilário, Sílvia Helena Henriques

**Affiliations:** 1 Universidade do Estado de Minas Gerais, Unidade Passos, Departamento de Enfermagem, Passos, MG, Brasil.; 2 Universidade de São Paulo, Escola de Enfermagem de Ribeirão Preto, Centro Colaborador da OPAS/OMS para o Desenvolvimento da Pesquisa em Enfermagem, Departamento de Enfermagem Geral e Especializada, Ribeirão Preto, SP, Brasil.; 3 Universidade do Estado de Minas Gerais, Unidade Passos, Departamento de Ciências Médicas, Passos, MG, Brasil.; 4 Universidade de São Paulo, Escola de Enfermagem de Ribeirão Preto, Centro Colaborador da OPAS/OMS para o Desenvolvimento da Pesquisa em Enfermagem, Ribeirão Preto, SP, Brasil.

**Keywords:** Treinamento por Simulação, Educação Interprofissional, Comunicação, Equipe de Assistência ao Paciente, Estudantes de Enfermagem, Educação em Enfermagem

## Abstract

aplicar um cenário de simulação realística para o desenvolvimento da comunicação e do trabalho em equipe em graduandos de enfermagem e analisar suas contribuições durante o estágio hospitalar.

estudo exploratório realizado com 27 estudantes do último período de enfermagem de uma instituição de ensino superior. Os dados foram coletados em 2021, utilizando-se um *checklist* e um cenário validado sobre comunicação e trabalho em equipe no ambiente hospitalar. A coleta ocorreu em duas etapas: inicialmente, durante a execução do cenário/*debriefing*, no laboratório de habilidades da instituição, e após o término do estágio hospitalar, por meio de entrevistas semiestruturadas. As discussões do *debriefing* e entrevistas foram analisadas por análise temática indutiva.

durante o *debriefing*, os alunos de graduação compartilharam seus pensamentos sobre a participação no cenário, a resolução do caso e suas experiências com comunicação e trabalho em equipe. Nas entrevistas, os alunos expressaram o desejo de incluir disciplinas adicionais nas aulas simuladas e destacaram os benefícios do uso da simulação na prática hospitalar.

a simulação proporcionou maior entendimento aos estudantes ao fomentar o raciocínio crítico-reflexivo sobre a comunicação e o trabalho em equipe durante o estágio hospitalar, evidenciando a importância de sua aplicação ainda durante a formação universitária.

## Destaques

**(1)** Aplicação de simulação clínica em estudantes antes da prática hospitalar. **(2)** Aprimoramento da comunicação e trabalho em equipe em estudantes de enfermagem. **(3)** Expandir conhecimentos utilizando atividade realista em laboratório. **(4)** Ampliação da visão do estudante sobre a atuação do enfermeiro no ambiente hospitalar. **(5)** Discussão sobre sentimentos e resolutividade de casos favorecida pelo *debriefing*.

## Introdução

Os enfermeiros, profissionais essenciais dos serviços de saúde, desempenham papel de relevância na assistência e respondem a esses serviços conforme suas competências^(1)^, ou seja, buscam aplicar seus conhecimentos, habilidades e atitudes no ambiente de trabalho. No contexto hospitalar, cenário de alta complexidade, esses profissionais têm buscado cada vez mais aprimorar suas competências, visando à colaboração interprofissional^(2)^.

A esse respeito, estudiosos acrescentam que a educação permanente e a formação voltada para a interprofissionalidade podem facilitar o desenvolvimento de competências que contribuem para a prática colaborativa^(3)^. Destaca-se que as competências interprofissionais englobam a clareza dos papéis e responsabilidades, os valores, a ética, a comunicação, o trabalho em equipe, o gerenciamento de conflitos, a liderança e a atenção em saúde focada no usuário, familiares e sociedade^(4-5)^. Desta forma, o estudo destaca duas competências essenciais à colaboração dos enfermeiros nos serviços de saúde: a comunicação e o trabalho em equipe. A comunicação é vista como um elemento crucial para a construção de um bom relacionamento interpessoal entre os cuidadores, usuários e família; já o trabalho em equipe permite a integração das diversas profissões e especialidades^(4)^.

Assim, visando garantir que os futuros enfermeiros aprimorem as competências de comunicação e trabalho em equipe durante a prática clínica, deve ser criado um espaço de ensino para esta finalidade^(6-7)^. Nesse contexto, a Educação Interprofissional destaca-se como estratégia efetiva para aquisição dessas competências. Esta abordagem, dentro das faculdades, visa envolver dois ou mais estudantes e/ou profissionais, incentivando-os a aprenderem juntos. Essa aprendizagem destina-se a prepará-los melhor para a cooperação nas instituições de saúde^(8)^.

Ainda, na contemporaneidade, sabe-se que os estudantes possuem grande afinidade com tecnologias interativas e imersivas e que são adeptos a usá-las como um recurso digital nativo. Essa familiaridade com a tecnologia pode servir como catalisador para a educação, pois, além de preparar novos trabalhadores de saúde, pode emergir como uma possibilidade de avançar na formação interprofissional e ainda auxiliar as instituições formadoras na inserção de metodologias ativas, como a simulação realística.

Neste contexto, o uso da simulação pode aprimorar as competências nos estudantes e permitir que eles reflitam sobre situações reais em um ambiente controlado, o que auxilia no campo prático^(9-10)^. Ainda, cenários de simulação são mais confiáveis quando elaborados e baseados em casos clínicos reais, pois podem possibilitar a reprodução de eventos que ocorrem no cotidiano de trabalho da equipe de enfermagem^(11)^.

Destarte, reconhece-se que a implementação de cenários clínicos validados para a formação profissional em saúde, que potencializem as competências interprofissionais em graduandos de enfermagem, é essencial, embora permaneçam subdesenvolvidas em muitas universidades no país. Diante disso, este estudo apresenta as seguintes questões norteadoras: Como pode ser desenvolvido um cenário de formação do profissional de saúde (simulação realística) para o desenvolvimento das competências interprofissionais de comunicação e trabalho em equipe durante a graduação do enfermeiro? Quais são os aspectos positivos e/ou limitantes da atividade simulada? Qual a percepção dos estudantes sobre o método utilizado? E quais contribuições dessa aprendizagem antecedem o estágio prático?

Neste ínterim, o estudo teve como objetivo aplicar um cenário de simulação realística para o desenvolvimento da comunicação e do trabalho em equipe em graduandos de enfermagem e analisar suas contribuições durante o estágio hospitalar.

## Método

### Delineamento do estudo

Estudo exploratório com abordagem qualitativa dos dados^(12)^. Para a escrita do artigo, foram utilizadas as recomendações do *Consolidated Criteria for Reporting Qualitative Studies* (COREQ), disponível na página da *web* da rede Equator^(13)^.

### Local do estudo

O estudo foi realizado no Laboratório de Habilidades de uma universidade pública, localizada no município de Passos, MG, Brasil. O laboratório é utilizado pelos estudantes de medicina, enfermagem, nutrição, estética e biomedicina. As aulas e atividades ministradas no local preparam o estudante para a prática clínica por meio do aprimoramento de competências. O laboratório dispõe de projetores, bancadas, leitos, simuladores de baixa fidelidade e posto de enfermagem.

### Período do estudo

A coleta de dados ocorreu entre os meses de maio e agosto de 2021.

### Participantes do estudo

Foram convidados 62 estudantes matriculados no Curso de Bacharelado em Enfermagem, sendo 22 no turno matutino e 40 no turno noturno. Foram selecionados os estudantes matriculados no décimo período do Curso de Bacharelado em Enfermagem, na disciplina de Estágio Curricular Supervisionado II. Os motivos de recusa para não participação do estudo (n=35), em sua grande maioria, eram profissionais (técnicos de enfermagem) e os indivíduos estavam, no período de coleta de dados, trabalhando em serviços de saúde na pandemia COVID-19, com turnos de trabalho alterados, limitando seu envolvimento na pesquisa. Dessa forma, participaram do estudo 27 graduandos de enfermagem.

### Variáveis do estudo

As variáveis demográficas eleitas para o estudo foram sexo, faixa etária, estado civil, filiação, naturalidade e profissão e as relacionadas a comunicação e trabalho em equipe foram fundamentadas nas diretrizes da *International Nursing Association Clinical Simulation & Learning* (INACSL): *Simulation-Enhanced Interprofessional Education* (Sim-IPE)^(14)^.

### Instrumentos utilizados para a coleta das informações

Foi utilizado um *checklist* e um cenário clínico validado que teve como foco as diretrizes da *International Nursing Association Clinical Simulation & Learning* (INACSL): *Simulation-Enhanced Interprofessional Education* (Sim-IPE)^(14)^. O *checklist* detalhando as ações do facilitador e dos estudantes participantes incluiu 28 itens subdividido em três domínios: *briefing* (7 itens); cenário em ação (14 itens) e *debriefing* (7 itens).

O cenário clínico, intitulado “Comunicação e trabalho em equipe no ambiente hospitalar”, teve como objetivo geral prestar cuidados de enfermagem a um paciente internado em uma unidade de Clínica Médica, desenvolvendo a comunicação assertiva e o trabalho em equipe frente a uma intercorrência (reação alérgica). O cenário clínico especificava os componentes cruciais para o seu progresso, tais como: o tempo de cada etapa da simulação (*Briefing*, Cenário em ação e *Debriefing*), a definição do caso clínico; os dados e a situação do paciente e as atividades que os estudantes deveriam executar. Além disso, especificava a área de atendimento e os personagens/atores (médico, paciente, acompanhante e enfermeiro do turno noturno) e os materiais e equipamentos.

Um outro instrumento de coleta de dados foi a entrevista semiestruturada individual, pautada em roteiro, contendo quatro questões: 1) Como foi participar de simulações clínicas durante a graduação em enfermagem? 2) Acredita que as simulações são importantes para o desenvolvimento de competências interprofissionais? Em que sentido? 3) O desenvolvimento do cenário simulado “Comunicação e trabalho em equipe no ambiente hospitalar” trouxe alguma contribuição para sua atuação durante o estágio prático? Se sim, quais? 4) Em quais situações e/ou locais você conseguiu desenvolver essas competências? Quais foram suas facilidades e dificuldades?

### Coleta de dados

A coleta de dados com os estudantes de enfermagem foi realizada em duas etapas. Primeiramente, foi conduzida uma atividade teórica sobre o tema e, na sequência, foi realizada a atividade de simulação clínica, com coleta de dados durante o *debriefing*. Posteriormente, após a conclusão da disciplina de Estágio Curricular Supervisionado II, foram realizadas entrevistas semiestruturadas individuais. A aula teórica teve duração máxima de vinte minutos e o desenvolvimento da simulação foi dividido em *briefing*: cinco minutos*;* cenário em ação: dez minutos e *debriefing*: vinte minutos, totalizando trinta e cinco minutos.

A pesquisadora principal, enfermeira, docente de enfermagem e com experiência na área, atuou como facilitadora nesse cenário. Para a realização do estudo, ela participou de disciplinas de pós-graduação sobre metodologias ativas e simulação realística e atuou como banca avaliadora de cenários simulados. Para a confiabilidade dos dados, um auxiliar de pesquisa, com mestrado em ciências da saúde e atuação como enfermeiro intensivista do hospital geral do município, foi convidado para participar do estudo. Esse auxiliar, que não tinha vínculo com a instituição de ensino, segundo os preceitos éticos, foi o responsável pelo convite aos estudantes de enfermagem para participar da pesquisa; acompanhou as atividades de simulação e, por fim, realizou registros e observações sobre os diálogos que os estudantes trouxeram durante o *debriefing*, auxiliando a pesquisadora principal.

Foram adotadas medidas para assegurar a integridade ética da pesquisa, como presença do auxiliar de pesquisa; anonimato e confidencialidade dos participantes; consentimento informado independente; separação e diferenciação entre pesquisador e avaliador e oportunidade de atuarem e exporem suas opiniões em ambientes neutros.

Os estudantes foram convidados a participar do estudo por meio de mensagens eletrônicas, como o *WhatsApp*. Após sua confirmação, eram orientados sobre os dias e horários da simulação no laboratório da instituição formadora citada anteriormente. Grupos com no máximo seis graduandos foram formados para participar da atividade simulada. A simulação foi realizada seis vezes, oferecendo diversas opções para a participação dos estudantes.

Durante a atividade de simulação clínica, no início, ou seja, no *Briefing*, foram realizadas as instruções iniciais da simulação; primeiramente, após o convite e escolha dos estudantes-atores, foi apresentado o ambiente em que o cenário deveria transcorrer, informando os objetivos e o caso clínico.

Na sequência, foi realizada a segunda etapa, Cenário em ação, em que foi apresentada uma situação para que os estudantes pudessem se apropriar da competência de comunicação e trabalho em equipe, utilizando-se de um contexto o mais próximo possível da realidade.

Para a operacionalização do cenário, foram recrutados dois estudantes para participarem como encenadores, um no papel de técnico de enfermagem e outro como enfermeiro. Os demais presentes permaneceram como expectadores. A facilitadora, após apresentar os materiais e equipamentos disponíveis no cenário, descreveu o caso para todos os participantes, tanto encenadores quanto expectadores e saiu da cena. Nesse momento, os atores/encenadores treinados (enfermeira do noturno, médico, acompanhante e paciente) iniciaram suas atuações.

No término dessa fase, os estudantes participaram do *Debriefing*. O pesquisador/facilitador convidou-os para se acomodarem em um semicírculo e incentivou que refletissem sobre o propósito da simulação; as emoções experimentadas ao longo do exercício; os aspectos positivos da atuação dos estudantes; as áreas que poderiam ser aprimoradas; as diferentes abordagens que poderiam ser adotadas durante a atividade, afastando críticas não construtivas e a interligação da teoria com a prática, com ênfase na comunicação e trabalho em equipe. Todos os envolvidos tiveram a oportunidade de compartilhar suas opiniões de forma equitativa.

Dando prosseguimento às atividades acadêmicas, os graduandos seguiram para o estágio curricular na área hospitalar. Após este período, foram convidados a participar da última fase da pesquisa, a entrevista semiestruturada foi agendada e conduzida de forma individual na instituição de ensino, em um local reservado, com duração aproximada de 20 minutos. As entrevistas foram realizadas apenas uma vez com cada participante. Todos os 27 estudantes participaram das entrevistas.

### Tratamento e análise dos dados

O material proveniente da etapa do *Debriefing* e das entrevistas individuais foi audiogravado e posteriormente transcrito. As transcrições foram feitas manualmente pelos pesquisadores responsáveis. Não foi utilizado *software* para interpretação dos dados.

Os dados foram analisados utilizando análise temática indutiva subdividida em seis fases: 1. Conhecimento dos dados; 2. Criação de códigos; 3. Identificação das temáticas; 4. Avaliação das temáticas; 5. Nomeação das temáticas; e 6. Elaboração do documento final^(15)^.

### Aspectos éticos

A pesquisa foi desenvolvida em conformidade com a Resolução 466/2012, e os participantes assinaram o Termo de Consentimento Livre e Esclarecido (TCLE). Com a finalidade de proteger as identidades dos envolvidos durante o *debriefing* e entrevistas, foram utilizadas como modo de identificação letras e números, tais como: E1, E2, E3, e assim sucessivamente. A pesquisa foi aprovada pelo Comitê de Ética e Pesquisa da Instituição Proponente sob protocolo CAAE 23555819.9.0000.5393 e da Instituição Coparticipante CAAE 23555819.9.3001.5112.

Destaca-se que os dados obtidos serão arquivados por cinco anos, sendo que o acesso será autorizado exclusivamente para os pesquisadores responsáveis e pelo CEP da Instituição Proponente e Coparticipante, caso seja solicitado. Após esse período, todos os registros supracitados serão apagados, de modo a evitar qualquer tipo de identificação dos participantes.

## Resultados

### Caracterização dos participantes

A Tabela 1 mostra as características dos participantes em relação às variáveis sexo, faixa etária, estado civil, filiação, naturalidade e profissão.

**Tabela 1 -:** Caracterização dos estudantes de enfermagem constituintes do estudo, segundo as variáveis demográficas (n = 27). Passos, MG, Brasil, 2024

**Variáveis**		**n**	**(%)**
Sexo	Feminino	24	89%
	Masculino	3	11%
Faixa etária	21 a 30 anos	22	82%
	31 a 40 anos	3	11%
	41 a 50 anos	2	7%
Estado civil	Solteiro	19	70%
	Casado	7	26%
	Divorciado	1	4%
Número de filhos	Sem filhos	19	70%
	1 filho	4	15%
	2 filhos	3	11%
	3 filhos	1	4%
Naturalidade	Passos	14	52%
	Outras cidades	13	48%
Profissão	Desempregado	14	52%
	Técnico de enfermagem	10	37%
	Outros	3	11%

Dos 27 (100%) graduandos em enfermagem, a maioria era do sexo feminino e na faixa etária entre 21 e 30 anos. No que diz respeito ao estado civil, 19 (70%) eram solteiros e sem filhos; 13 (48%) participantes residiam em outras cidades. Ressalta-se que 10 (37%) dos participantes eram técnicos de enfermagem, atuantes na Santa Casa de Passos e desses, quatro atuavam entre um e cinco anos na instituição, três entre seis e dez anos e outros três atuavam há mais de 11 anos. Por fim, três participantes responderam que exerciam outra profissão, sendo recepcionistas e manicures.

Os resultados permitiram identificar duas grandes categorias temáticas: cenário simulado para o desenvolvimento da comunicação e trabalho em equipe em graduandos de enfermagem e contribuições da simulação para a prática clínica.

### Cenário simulado para o desenvolvimento da comunicação e trabalho em equipe em graduandos de enfermagem

#### Em relação à experiência simulada

Para a simulação foi planejado o seguinte cenário: passagem de plantão no posto de enfermagem pela enfermeira do noturno aos estudantes-atores (enfermeiro e técnico de enfermagem): paciente idosa, hipertensa, com período de confusão, internada há menos de duas horas, com quadro de pneumonia, com pico febril de 37,9°C e medicada com 40 gotas de dipirona via oral. A filha chega na enfermaria com os medicamentos de uso contínuo da mãe e aperta a campainha. Os estudantes, ao verificarem que a campainha da enfermaria estava acionada, foram até a paciente. Ao chegar na enfermaria, a filha da paciente questiona os estudantes sobre a hiperemia e prurido na face, nos braços e no tórax que a paciente apresentava. Na sala de prescrição, encontrava-se o médico responsável pelo paciente, que estava avaliando os prontuários para realizar a visita médica.

Nesse contexto, os estudantes, com os conhecimentos adquiridos tanto nas disciplinas anteriormente cursadas na graduação, assim como pelo bloco teórico do Estágio Supervisionado oferecido antes da atividade de simulação, deveriam avaliar a situação; oferecer os cuidados de enfermagem necessários e imprescindíveis conforme sua avaliação; promover comunicação clara e efetiva com o usuário, seus familiares e equipe e colaborar para a resolução do problema apresentado pelo usuário, buscando utilizar seus conhecimentos, habilidades e atitudes em uma eventualidade que pode acontecer no contexto do hospital.

#### Debriefing

Ao término da atividade simulada, os estudantes foram convidados a participar do *Debriefing* e puderam refletir sobre as situações em que estiveram envolvidos e expressar seus sentimentos, analisar os pontos positivos de sua atuação e apontar o que fariam de diferente durante a simulação. Dessa forma, tendo como foco a análise temática indutiva, em que os códigos e temas gerados emergem dos próprios relatos apresentados pelos estudantes, foi possível identificar novas percepções, sentimentos, conhecimentos e pontos de melhoria durante a simulação:

*A gente veio e eu não imaginei que teria uma situação assim para atuar, na hora fiquei meio nervosa, sem saber o que fazer.* (E20)

*Fiquei com um pouco de receio no início, de ser uma coisa complicada, mas não foi.* (E1)

Ao mesmo tempo, os estudantes mostr1aram satisfação em participar da experiência simulada:

*Eu amei! É de grande valia aprender isso aqui e depois poder aplicar na prática, a gente pode até ficar um pouco nervoso, só que na hora que chegar no hospital nós vamos lembrar do que aconteceu aqui, ‘agora eu faço isso, isso e isso’, conforme eu aprendi na aula.* (E13)

Nesse ínterim, os participantes discorreram sobre a aproximação do cenário da realidade prática, auxiliando na aquisição de conhecimentos:

*Foi bom porque a gente fica mais perto do real, da prática, por mais que a gente não esteja no hospital, nós somos cobrados como se estivéssemos lá, então a gente acaba prestando mais atenção aos detalhes, fica mais próximo do real.* (E26)

*Na simulação, além de ter essa proximidade com a realidade, eu vejo que nós podemos pensar mais fora da caixa; nós discutimos com as outras pessoas, vemos se eles pensam ou agem de forma diferente do que a gente esperava ou aprende nos livros.* (E24)

A partir desse contexto, os participantes puderam refletir sobre os pontos positivos vivenciados no cenário, como aspectos relacionados à resolutividade do problema apresentado, o acolhimento do usuário, a comunicação e o trabalho em equipe, como apresentado na sequência:

*Eu senti que minha atuação foi adequada para a situação; a gente identificou o problema assim que a gente adentrou no quarto e nós solicitamos avaliação médica, então, para aquela situação específica, era o indicado a se fazer.* (E10)

*A questão de acolher o paciente, o acompanhante, acho que nós acolhemos muito bem.* (E1)

*Eu também achei que teve a comunicação rápida com a médica, com os profissionais que eram responsáveis naquele momento.* (E26)

*Acho que nós trabalhamos bem em equipe, as duas bem integradas com a médica.* (E2)

No entanto, os estudantes também levantaram pontos negativos/limitantes que dificultaram a assistência ao usuário, sendo estimulados a expressarem se fariam algo distinto do que foi realizado e de que maneira poderiam aprimorar suas ações. Os relatos mostraram aspectos voltados para a melhoria da investigação da queixa do usuário, a realização do exame físico completo, a segurança na sua identificação e a apresentação pessoal:

*Deveria ter descoberto a paciente para avaliar o prurido do corpo... elas perguntaram se estava sentindo mais alguma coisa, mas não avaliaram.* (E23)

*Eles deveriam desde o começo ter colocado na identificação da paciente que ela era alérgica, devemos sempre grifar a alergia do paciente!* (E12)

*Deveriam ter se apresentado também, primeira coisa que faz quando chega no quarto: ‘eu sou fulano’.* (E12)

Esses pontos supracitados foram bastante discutidos durante o *Debriefing*, relacionando a teoria com a prática vivenciada durante a simulação.

### Contribuições da simulação para a prática clínica

Na última etapa da pesquisa, após a imersão dos estudantes em campo prático, os 27 estudantes foram convidados a participar de entrevistas individuais, realizadas pela própria pesquisadora principal. Os discentes identificaram que a participação no cenário simulado, previamente à realização das imersões em campo de estágio na área hospitalar, trouxe embasamento para suas ações durante a prática clínica. Dessa forma, novos códigos e temas foram gerados a partir das entrevistas.

Os estudantes relataram melhor comunicação e trabalho em equipe, e assim sugeriram a inclusão de mais simulações na grade curricular, como é apresentado nos relatos na sequência:

*Primeiro eu acho que deveria ter mais simulações, porque se pegasse o estudante e levasse ele da teoria para uma simulação no laboratório corrigiria seus erros; e depois levasse ele para a prática, eu acho que nós teríamos enfermeiros muito mais capacitados.* (E11)

*Eu acho que os professores deveriam fazer mais simulações em laboratório, ajudaria muito o estudante, ficaria bem mais interessante; os estudantes aprenderiam mais, quebraria as barreiras.* (E5)

Na sequência, os estudantes referiram os benefícios do cenário durante sua imersão no campo de estágio:

*Todos os pontos falhos e trabalhados durante a simulação, eu comecei a prestar mais atenção no estágio, então foi mais fácil, aprender a linguagem corporal, falar com mais calma, olhar no olho, ver se a pessoa entendeu realmente o que você quis falar, então eu melhorei nisso.* (E2)

*Teve um dia do estágio que os colegas esqueceram de se apresentar e aí me lembrou muito a simulação, eu me atentei sobre isso.* (E26)

*Apliquei na diálise, melhorou minha comunicação e a passagem de plantão, porque às vezes fica algumas coisas falhas. Hoje eu consigo dar uma maior atenção aos acompanhantes, ficou bem mais fácil.* (E5)

*A simulação me ajudou muito porque antes nós não trabalhávamos juntos, era trabalho individual, então depois desse estágio a gente se ajudou mais e a comunicação foi bem melhor.* (E14)

Por fim, os estudantes apontaram as contribuições para a aprendizagem enquanto futuros profissionais atuantes no mercado de trabalho:

*Vai me ajudar muito porque você tem uma outra visão sobre o trabalho em equipe, sobre o jeito de comunicar, de falar com a equipe médica; então acaba ajudando a gente em vários pontos, como líder mesmo, tomar frente da situação e poder liderar com segurança e transmitir essa segurança para a equipe.* (E18)

*Eu acho que vai me ajudar bastante porque o trabalho em equipe e a comunicação é o principal da enfermagem, é tudo o que precisa para chegar no paciente; não tem como, sem comunicação é impossível! Acredito que eu vou ter mais segurança para atuar como enfermeiro, para realizar meu trabalho.* (E23)

## Discussão

Este estudo investigou uma experiência simulada com graduandos de enfermagem visando desenvolver as competências de comunicação e trabalho em equipe. Os resultados elucidaram a implementação da simulação realística com graduandos de enfermagem como fundamental para a compreensão e aprimoramento das competências, comunicação e o trabalho em equipe, essenciais para a práxis profissional e colaborativa no contexto hospitalar.

Evidenciou-se que os estudantes puderam desenvolver as competências desde o primeiro contato com o ambiente da simulação: realizaram a comunicação e o trabalho em equipe com o enfermeiro do plantão noturno, com o paciente e acompanhante, com o médico e identificaram quadro alérgico da paciente que necessitava de resolução imediata. A enfermagem exige uma interação eficaz entre a equipe e o usuário, e a simulação proporciona um ambiente controlado e seguro para que os estudantes vivenciem situações reais, aprendam a gerenciar conflitos, melhorem a assertividade na comunicação e fortaleçam a cooperação interprofissional^(16)^.

No *debriefing*, os relatos revelaram ainda emoções, sentimentos e percepções da aprendizagem dos estudantes durante a vivência da experiência simulada, o que pode auxiliar na discussão para outras simulações e/ou pesquisas, bem como contribuir para reflexão de educadores para que possam ter a oportunidade de enriquecer ou replicar tal atividade. Estudiosos afirmam que a simulação clínica tem se tornado uma das abordagens mais utilizadas no ensino em enfermagem, pois auxilia os discentes a obterem mais conhecimento e confiança para agir, além de realizar o aprofundamento teórico por meio de uma cena^(16)^.

Embora as atividades simuladas contribuam para o aprimoramento de habilidades, atitudes e conhecimento, é reconhecido que elas também têm o potencial de gerar temor, ansiedade e incerteza^(17)^, como foi possível identificar certa ansiedade nos estudantes neste estudo. Dessa forma, é importante que estudantes estejam familiarizados com a proposta da simulação, com os objetivos, com o ambiente simulado e com as tecnologias disponíveis. Cabe ao professor nesse momento sensibilizá-los para a troca de vivências e construção de novos conhecimentos^(18)^.

Ao mesmo tempo, a aproximação entre a simulação e a realidade prática traz boa receptividade do estudante a essa metodologia^(19)^, pois pode transportar o estudante a situações que se assemelham à realidade, em que é permitido errar, repetir processos, discutir sobre a assistência ao paciente de forma segura e proporcionam uma formação mais sólida ao integrar teoria e prática^(20)^. Assim, durante a simulação, “o aprender sobre, com e uns com os outros” pode possibilitar uma prática colaborativa e uma assistência eficaz, como é preconizado pela Educação Interprofissional^(14)^.

Concomitante a isso, as unidades contextuais também revelaram falas, as quais mostraram que os estudantes refletiram sobre quais atitudes poderiam ter sido realizadas de maneira diferente; revelaram a ausência de algumas ações importantes que foram compreendidas como limitações de suas participações no cenário, a exemplo de investigação da queixa do usuário, realização do exame físico completo, segurança na identificação correta e apresentação pessoal.

Sabe-se que o crescimento do estudante considera aspectos que podem ser aprimorados, levando à identificação de possíveis deficiências em seu aprendizado. Isso ressalta a importância da simulação^(21)^, juntamente com a inclusão de diferentes situações e abordagens, ainda nos primeiros anos de graduação, que pode incentivar os estudantes a discutirem melhor as condutas a serem tomadas com o paciente, familiares e equipe profissional. Ademais, o retorno/*feedback* do docente é crucial e possibilita a reflexão dos estudantes sobre suas práticas e comportamentos profissionais^(22)^. Desse modo, o uso da simulação trouxe contribuições essenciais no aprendizado dos discentes ao incentivar o pensamento crítico e reflexivo, ampliando sua consciência e corresponsabilidade em aprimorar as competências exigidas na prática profissional, como a comunicação e o trabalho em equipe.

Portanto, os graduandos, ao terem oportunidade de vivenciar a simulação, antecedendo o estágio prático, são capazes de aprimorar seus conhecimentos e habilidades, de refletir sobre o seu papel profissional e de aprender a trabalhar colaborativamente, contribuindo significativamente para a formação de sua identidade profissional^(23)^. Ao mesmo tempo, estudos^(24-25)^ demonstram que os estudantes que participaram de atividades simuladas absorveram mais conhecimento do que aqueles que não participaram. Isso destaca a importância de realizar treinamentos/capacitações de forma regular, para garantir a retenção eficaz de conhecimentos em longo prazo. Para isso, faz-se necessário um corpo docente capacitado.

Pesquisadores descrevem que a logística e o financiamento constituem o principal obstáculo para a adoção de atividades simuladas nas instituições educacionais^(26)^. Outros argumentam que a introdução da simulação clínica é um desafio para os educadores, pois demanda tempo e esforço para a capacitação dos docentes, bem como a preparação, definição dos objetivos de aprendizagem e execução da simulação^(27)^.

Entretanto, cenários simulados estão intrinsecamente ligados à capacidade criativa e ao empenho do docente, tanto na sua elaboração quanto na sua implementação, servindo como um recurso valioso do ensino, desde que embasado nos princípios teóricos^(28)^. Dessa forma, é imprescindível inserir debates nos projetos políticos-pedagógicos dos cursos sobre a Educação Interprofissional e a utilização da simulação realística, visando preparar adequadamente os docentes e os futuros profissionais de enfermagem para a colaboração nos serviços de saúde.

O cenário deste estudo apresenta um caso/evento do ambiente hospitalar, o que pode limitar sua utilização em outras áreas, como na saúde pública, mesmo sabendo que a comunicação e o trabalho em equipe são fundamentais em qualquer local de trabalho.

Este estudo contribui para o avanço do saber científico, uma vez que oferece aos cursos de graduação em enfermagem a oportunidade de explorar e desenvolver a comunicação e o trabalho em equipe em discentes, por meio de um cenário validado. Assim, os mesmos podem expandir seus conhecimentos utilizando uma atividade realista desenvolvida em laboratório. Ademais, a simulação pode auxiliar os estudantes no desenvolvimento de uma visão mais abrangente sobre a atuação do enfermeiro no ambiente hospitalar, guiando esse futuro profissional para uma prática segura e colaborativa.

## Conclusão

Com o intuito de educar e desenvolver/aprimorar as competências de comunicação e trabalho em equipe nos graduandos em enfermagem para uma prática interprofissional e colaborativa, foi aplicado um cenário simulado sobre um paciente internado em uma unidade de clínica médica no qual o discente teve a oportunidade de aplicar essas competências ante um evento inesperado. Desse modo, a simulação proporcionou um maior entendimento aos estudantes ao incentivar a análise crítica e reflexiva sobre a comunicação e o trabalho em equipe durante o estágio hospitalar, destacando a relevância de sua utilização ainda na formação universitária.

# Translation: Simulación clínica para el desarrollo de la comunicación y el trabajo en equipo en enfermería*

**DOI:** 10.1590/1518-8345.7560.4547

**Authors:** Aline Teixeira Silva, Laura Andrian Leal, Maria Inês Lemos Coelho Ribeiro, Monise Martins da Silva, Jeniffer Stephanie Marques Hilário, Sílvia Helena Henriques

**Affiliations:** 1 Universidade do Estado de Minas Gerais, Unidade Passos, Departamento de Enfermagem, Passos, MG, Brasil.; 2 Universidade de São Paulo, Escola de Enfermagem de Ribeirão Preto, Centro Colaborador de la OPS/OMS para el Desarrollo de la Investigación en Enfermería, Departamento de Enfermagem Geral e Especializada, Ribeirão Preto, SP, Brasil.; 3 Universidade do Estado de Minas Gerais, Unidade Passos, Departamento de Ciências Médicas, Passos, MG, Brasil.; 4 Universidade de São Paulo, Escola de Enfermagem de Ribeirão Preto, Centro Colaborador de la OPS/OMS para el Desarrollo de la Investigación en Enfermería, Ribeirão Preto, SP, Brasil.

**Keywords:** Entrenamiento Simulado, Educación Interprofesional, Comunicación, Grupo de Atención al Paciente, Estudiantes de Enfermería, Educación en Enfermería

## Abstract

aplicar un escenario de simulación realista para desarrollar la comunicación y el trabajo en equipo en estudiantes de enfermería y analizar sus contribuciones durante la práctica profesional hospitalaria.

estudio exploratorio realizado con 27 estudiantes del último período de enfermería de una institución de educación superior. Los datos fueron recolectados en 2021, mediante una lista de verificación y un escenario validado sobre comunicación y trabajo en equipo en el ambiente hospitalario. La recolección ocurrió en dos etapas: primero, durante la ejecución del escenario/*debriefing*, en el laboratorio de habilidades de la institución, y después del final de la práctica profesional hospitalaria, a través de entrevistas semiestructuradas. Las discusiones del *debriefing* y las entrevistas se analizaron mediante análisis temático inductivo.

durante el *debriefing*, los estudiantes de pregrado compartieron sus pensamientos sobre la participación en el escenario y la resolución del caso y sus experiencias con la comunicación y el trabajo en equipo. En las entrevistas, los estudiantes expresaron el deseo de incluir asignaturas adicionales en clases simuladas y destacaron los beneficios del uso de la simulación en la práctica hospitalaria.

la simulación proporcionó mayor comprensión a los estudiantes al fomentar el razonamiento crítico-reflexivo sobre la comunicación y el trabajo en equipo durante la práctica profesional hospitalaria, resaltando la importancia de su aplicación incluso durante la formación universitaria.

## Destacados

**(1)** Aplicación de simulación clínica en estudiantes antes de la práctica hospitalaria. **(2)** Mejorar la comunicación y el trabajo en equipo en estudiantes de enfermería. **(3)** Ampliar conocimientos utilizando actividades de laboratorio realistas. **(4)** Ampliar la visión del estudiante sobre el rol de la enfermera en el ámbito hospitalario. **(5)** Discusión sobre sentimientos y resolución de casos favorecidos por el *Debriefing*.

## Introducción

Los enfermeros, profesionales de los servicios esenciales de salud, cumplen un rol relevante en la asistencia y responden a esos servicios de acuerdo con sus competencias^(1)^, es decir, buscan aplicar sus conocimientos, habilidades y aptitudes en el ambiente de trabajo. En el contexto hospitalario, escenario de alta complejidad, estos profesionales intentan cada vez más optimizar sus habilidades, buscando la colaboración interprofesional^(2)^.

En este sentido, estudios afirman que la educación y la formación continua orientada a la interprofesionalidad pueden facilitar el desarrollo de habilidades que contribuyan a la práctica colaborativa^(3)^. Se destaca que las competencias interprofesionales abarcan claridad de roles y responsabilidades, valores, ética, comunicación, trabajo en equipo, manejo de conflictos, liderazgo y atención de la salud enfocada en los usuarios, sus familiares y en la sociedad^(4-5)^. De este modo, el estudio destaca dos habilidades esenciales para la colaboración de los enfermeros en los servicios de salud: la comunicación y el trabajo en equipo. La comunicación es vista como un elemento crucial para construir una buena relación interpersonal entre cuidadores, usuarios y familiares. El trabajo en equipo permite la integración de diferentes profesiones y especialidades^(4)^.

En ese marco, para lograr que los futuros enfermeros mejoren sus habilidades de comunicación y trabajo en equipo durante la práctica clínica, se debe crear un espacio docente para tal fin^(6-7)^. En este contexto, la Educación Interprofesional destaca como una estrategia eficaz para la adquisición de estas habilidades. Este enfoque, dentro de las facultades, tiene como objetivo implicar a dos o más estudiantes y/o profesionales e incentivarlos a aprender juntos. El aprendizaje tiene como objetivo prepararlos mejor para la cooperación en instituciones de salud^(8)^.

Además, en la actualidad, se sabe que los estudiantes tienen una gran afinidad con las tecnologías interactivas e inmersivas y que son expertos en utilizarlas como recurso digital nativo. Esta familiaridad con la tecnología puede servir como catalizador de la educación, ya que, además de preparar nuevos trabajadores de la salud, puede surgir como una posibilidad para avanzar en la formación interprofesional y contribuir con las instituciones de formación en la inserción de metodologías activas, como la simulación realista.

En este contexto, el uso de la simulación puede mejorar las habilidades de los estudiantes y permitirles reflexionar sobre situaciones reales en un ambiente controlado, favoreciéndolos en el campo práctico^(9-10)^. Además, los escenarios de simulación son más confiables cuando son desarrollados y basados en casos clínicos reales, dado que permiten la reproducción de eventos que ocurren en el trabajo diario del equipo de enfermería^(11)^.

Por lo tanto, se reconoce que la implementación de escenarios clínicos validados para la formación profesional en salud, que potencien las habilidades interprofesionales en los egresados de enfermería, es fundamental, aunque aún se encuentren poco desarrollados en muchas universidades del país. Por lo tanto, este estudio presenta las siguientes preguntas orientadoras: ¿Cómo se puede desarrollar un escenario de formación de profesionales de la salud (simulación realista) para desarrollar habilidades de comunicación interprofesional y trabajo en equipo durante la graduación en enfermería? ¿Cuáles son los aspectos positivos y/o limitantes de la actividad simulada? ¿Cuál es la percepción de los estudiantes sobre el método utilizado? ¿Y qué aportes de este aprendizaje preceden a la práctica profesional hospitalaria?

En ese marco, el estudio tuvo como objetivo aplicar un escenario de simulación realista para el desarrollo de la comunicación y el trabajo en equipo en estudiantes de enfermería y analizar sus contribuciones durante la práctica profesional hospitalaria.

## Método

### Diseño del estudio

Estudio exploratorio con enfoque cualitativo de datos^(12)^. Para la redacción del artículo se utilizaron las recomendaciones del *Consolidated Criteria for Reporting Qualitative Studies* (COREQ), disponible en el sitio *web* de la red Equator^(13)^.

### Sede del estudio

El estudio se llevó a cabo en Laboratorio de Habilidades de una universidad pública, ubicada en la ciudad de Passos, MG, Brasil. El laboratorio es utilizado por los estudiantes de medicina, enfermería, nutrición, estética y biomedicina. Las clases y actividades realizadas en el sitio preparan al estudiante para la práctica clínica perfeccionando sus habilidades. El laboratorio cuenta con proyectores, mesas, camas, simuladores de baja fidelidad y puesto de enfermería.

### Período del estudio

La recolección de datos se realizó entre mayo y agosto de 2021.

### Participantes del estudio

Se invitó a 62 estudiantes matriculados en la carrera de Licenciatura en Enfermería, 22 del turno matutino y 40 del turno vespertino. Fueron seleccionados los estudiantes matriculados en el décimo periodo de la Licenciatura en Enfermería, en la disciplina Práctica Curricular Supervisada II. Los motivos de negativa a participar en el estudio (n=35), en su gran mayoría, fueron profesionales (técnicos de enfermería) y los individuos se encontraban, durante el período de recolección de datos, trabajando en servicios de salud durante la pandemia de COVID-19, con turnos de trabajo alterados, lo que limitó su participación en la investigación. Así, participaron en el estudio 27 estudiantes de enfermería.

### Variables de estudio

Las variables demográficas elegidas para el estudio fueron sexo, grupo de edad, estado civil, filiación, lugar de nacimiento y profesión y las relacionadas con la comunicación y el trabajo en equipo se basaron en los lineamientos de la *International Nursing Association Clinical Simulation & Learning* (INACSL): *Simulation-Enhanced Interprofessional Education* (Sim-IPE)^(14)^.

### Instrumentos utilizados para recopilar información.

Se utilizó una lista de verificación y un escenario clínico validado, con foco en la *International Nursing Association Clinical Simulation & Learning* (INACSL): *Simulation-Enhanced Interprofessional Education* (Sim-IPE)^(14)^.

La lista de verificación*,* que describía las acciones del facilitador y los estudiantes participantes, constaba de 28 elementos distribuidos en 3 dominios: *briefing* (7 elementos); escenario en acción (14 ítems) y *debriefing* (7 ítems).

El escenario clínico denominado “Comunicación y trabajo en equipo en el ambiente hospitalario”, tuvo como objetivo general brindar atención de enfermería a un paciente ingresado en una unidad de Clínica Médica, desarrollando la comunicación asertiva y el trabajo en equipo ante un incidente (reacción alérgica). Al mismo tiempo, el escenario delineó los componentes elementales para su avance, tales como: el tiempo de cada etapa de la simulación (*Briefing*, Escenario en acción y *Debriefing*), la definición del caso clínico; los datos y situación del paciente; las actividades que deben realizar los estudiantes; además, especificó el área de servicio y los personajes/actores (médico, paciente, acompañante y enfermero nocturno) y los materiales y equipos.

Para las entrevistas, luego de la aplicación del escenario, se utilizó un guion previamente elaborado, con cuatro preguntas: 1) ¿Cómo fue participar en simulaciones clínicas durante tus estudios de grado? 2) ¿Crees que las simulaciones son importantes para el desarrollo de habilidades interprofesionales? ¿En qué sentido? 3) ¿El desarrollo del escenario simulado “Comunicación y trabajo en equipo en el entorno hospitalario” aportó contribuciones a tu desempeño durante las prácticas? Si es así, ¿cuáles? 4) ¿En qué situaciones y/o lugares pudiste desarrollar estas habilidades? ¿Cuáles fueron sus ventajas y dificultades?

### Recopilación de datos

La recolección de datos con los estudiantes se realizó en dos etapas. Primero se realizó una actividad teórica sobre el tema y, en seguida, se llevó a cabo la actividad de simulación clínica, con recolección de datos durante el *debriefing*. Con posterioridad, después de finalizar la materia Práctica Curricular Supervisada II, se efectuaron entrevistas individuales semiestructuradas, con el objetivo de analizar las repercusiones del escenario en la práctica hospitalaria del estudiante. La clase teórica tuvo una duración máxima de veinte minutos y el desarrollo de la simulación se dividió en *un briefing:* cinco minutos*;* escenario en acción: diez minutos y *debriefing*: veinte minutos, lo que totalizó treinta y cinco minutos.

La investigadora principal, enfermera, profesora de enfermería con experiencia en el área, actuó como facilitadora en este escenario. Para la realización del estudio participó en cursos de posgrado sobre metodologías activas y simulación realista y conformó el panel de evaluación de escenarios simulados. Al mismo tiempo, para una mayor confiabilidad de los datos, se invitó a participar en el estudio a un asistente de investigación, que tiene una maestría en ciencias de la salud y trabaja como enfermero de cuidados intensivos en el hospital general de la ciudad. Cabe señalar que este asistente no tenía vinculación con la institución y siguió todos los preceptos éticos: invitó a los estudiantes a realizar la investigación; siguió todas las actividades de simulación y, finalmente, realizó registros y observaciones sobre los diálogos que los estudiantes aportaron durante el *debriefing*, asistiendo al investigador principal.

A fin de garantizar la integridad ética de la investigación, se estableció: presencia del asistente de la investigación; anonimato y confidencialidad de los participantes; consentimiento informado independiente; separación y diferenciación entre investigador y evaluador y asegurar la oportunidad de actuar y expresar sus opiniones en ambientes neutrales.

Los estudiantes fueron invitados a participar en el estudio a través de mensajes electrónicos en aplicaciones como *WhatsApp*. Luego de la confirmación, fueron instruidos sobre los días y horarios de la simulación en el laboratorio de la institución formadora mencionada anteriormente. Se formaron grupos con un máximo de seis estudiantes universitarios para participar en la actividad simulada. La simulación se realizó seis veces, ofreciendo varias opciones para la participación de los estudiantes. Es de destacar que los estudiantes que no participaron del estudio (n=35), en su gran mayoría, eran profesionales (técnicos en enfermería) que se encontraban trabajando, en el turno respectivo, en servicios de salud en el combate a la pandemia de COVID-19, habiendo sufrido cambios en sus horarios de trabajo que limitaron su participación en la investigación.

Durante la actividad de simulación clínica, al comienzo, es decir en el *Briefing*, se realizaron las instrucciones iniciales de simulación. En primer lugar, luego de invitar y elegir a los estudiantes-actores, se presentó el ambiente en el que debería desarrollarse el escenario, y se informó los objetivos y el caso clínico. A continuación, se llevó a cabo la segunda etapa, Escenario en acción, en la que se presentó una situación para que los estudiantes adquirieran habilidades de comunicación y trabajo en equipo, utilizando un contexto lo más cercano posible a la realidad.

Para ejecutar el escenario, se reclutó a dos estudiantes para que representaran la escena, uno en el rol de técnico de enfermería y el otro como enfermero. El resto de los presentes permanecieron como espectadores. La facilitadora, tras presentar los materiales y equipos disponibles en el lugar, pasó el caso a todos los participantes, tanto actores como espectadores, y abandonó la escena. En ese momento, los actores/personajes capacitados (enfermera del turno nocturno, médico, acompañante y paciente) comenzaron sus actuaciones. Al final de esta etapa, los estudiantes participaron en el *Debriefing*. El investigador/facilitador los invitó a sentarse en semicírculo y los animó a reflexionar sobre: el propósito de la simulación; las emociones vividas a lo largo del ejercicio; los aspectos positivos del desempeño estudiantil; las áreas que se podrían perfeccionar; los diferentes enfoques que se podrían adoptar durante la actividad, evitando críticas no constructivas; y la interconexión de la teoría con la práctica, con énfasis en la comunicación y el trabajo en equipo. Todos los involucrados tuvieron la oportunidad de compartir sus opiniones por igual.

Para dar continuidad a sus actividades académicas, los estudiantes realizaron prácticas en el ámbito hospitalario. Luego de este período, fueron invitados a participar en la última etapa de la investigación, la entrevista semiestructurada. La entrevista fue programada y se llevó a cabo de manera individual en la institución educativa, en un espacio privado, con una duración aproximada de 20 minutos. Las entrevistas se realizaron una sola vez con cada participante. Los 27 estudiantes participaron en las entrevistas, sin saturación de datos.

### Procesamiento y análisis de datos

Todo el material de la etapa *de Debriefing* y de las entrevistas individuales fue grabado en audio y luego transcrito. Las transcripciones se realizaron manualmente, con intervención exclusiva de los investigadores responsables. No se utilizó *software* para la interpretación de datos.

Los datos se analizaron mediante análisis temático inductivo. El análisis temático se dividió en seis etapas, a saber: 1. Conocimiento de los datos; 2. Creación de códigos; 3. Identificación de temas; 4. Evaluación de temas; 5. Denominación de temas; y 6. Elaboración del documento final^(15)^.

### Aspectos éticos

La investigación se desarrolló de acuerdo con la Resolución 466/2012 y los participantes firmaron el Formulario de Consentimiento Libre e Informado (TCLE). Para proteger las identidades de los implicados durante el *debriefing* y las entrevistas, se utilizaron letras y números como medio de identificación, tales como: E1, E2, E3, etc. La investigación fue aprobada por el Comité de Ética e Investigación de la Institución Proponente bajo protocolo CAAE 23555819.9.0000.5393 y la Institución Coparticipante CAAE 23555819.9.3001.5112.

Es pertinente resaltar que los datos obtenidos serán archivados por cinco años, y el acceso será autorizado exclusivamente a los investigadores responsables y al CEP de la Institución Proponente y Coparticipante, si así se solicita. Transcurrido este plazo, todos los registros mencionados anteriormente serán eliminados, con el fin de evitar cualquier tipo de identificación de los participantes.

## Resultados

### Caracterización de los participantes.

La Tabla 1 muestra las características de los participantes en relación con las variables: género, grupo etario, estado civil, descendientes, lugar de nacimiento y profesión.

**Tabla 1 -:** Perfil sociodemográfico de los estudiantes de enfermería incluidos en el estudio (n = 27). Passos, MG, Brasil, 2024

**Variables**	**Caracterización**	**n**	**(%)**
Género	Femenino	24	89%
	Masculino	3	11%
Grupo etario	21 a 30 años	22	82%
	31 a 40 años	3	11%
	41 a 50 años	2	7%
Estado civil	Soltero	19	70%
	Casado	7	26%
	Divorciado	1	4%
Descendientes	0 hijos	19	70%
	1 hijo	4	15%
	2 hijos	3	11%
	3 hijos	1	4%
Lugar de nacimiento	Passos	14	52%
	Otras ciudades	13	48%
Profesión	Desempleados	14	52%
	Técnico en enfermería	10	37%
	Otras	3	11%

De los 27 graduados en enfermería, la mayoría eran mujeres y tenían entre 21 y 30 años. En cuanto al estado civil, 19 (70%) eran solteros y sin hijos. Trece (48%) participantes vivían en otras ciudades y 11 (79%) de ellos realizaron pasantías no obligatorias (becarios) en diversas instituciones de salud de la ciudad. También se evidenció que diez participantes eran técnicos de enfermería y trabajaban en la Santa Casa de Passos. De ellos, cuatro habían trabajado en la institución entre 1 y 5 años, tres entre 6 y 10 años y otros tres habían trabajado por más de 11 años. Finalmente, tres participantes respondieron que tenían otra profesión, y trabajaban como recepcionistas y manicuristas.

Los resultados permitieron identificar dos grandes categorías temáticas: escenario simulado para el desarrollo de la comunicación y trabajo en equipo en estudiantes de enfermería y contribuciones de la simulación a la práctica clínica.

### Escenario simulado para el desarrollo de la comunicación y el trabajo en equipo en estudiantes de enfermería

#### Respecto a la experiencia simulada

Para la simulación se planteó el siguiente escenario: realizó el pase de guardia en el puesto de enfermería por parte de la enfermera del turno nocturno a los estudiantes-actores (enfermero y técnico de enfermería): paciente anciana, hipertensa, con período de confusión, hospitalizada por menos de dos horas, con neumonía; Tuvo un pico febril de 37,9° C y fue tratada con 40 gotas de dipirona por vía oral. La hija acababa de llegar con los medicamentos que su madre estaba tomaba y presionó el timbre. Los estudiantes, al percatarse de que sonaba el timbre de la habitación, se dirigieron hacia la paciente. En ese momento, la hija de la paciente cuestionó a los estudiantes sobre la hiperemia y picazón local que presentaba su madre. En la sala de recetas se encontraba el médico responsable de la paciente, quien evaluaba la historia clínica para realizar la visita médica.

En este contexto, los estudiantes, con los conocimientos adquiridos tanto en las materias previamente cursadas durante sus estudios de pregrado, como en el bloque teórico que se ofreció antes del escenario, deben evaluar la situación; ofrecer los cuidados de enfermería necesarios e imprescindibles según su valoración; promover una comunicación clara y efectiva con el usuario, su familia y el equipo; colaborar para resolver el problema que presenta el usuario, y buscar formas para utilizar sus conocimientos, habilidades y actitudes ante cualquier eventualidad que pueda presentarse en el contexto hospitalario.

#### Debriefing

Al finalizar la actividad simulada, los estudiantes fueron invitados a participar en el *Debriefing*. Pudieron reflexionar sobre las situaciones en las que se vieron involucrados en la simulación y expresar sus sentimientos, analizar los puntos positivos de su desempeño y señalar qué harían diferente durante la simulación. De esta manera, a partir del análisis temático inductivo, en el que los códigos y temas generados emergen de los informes presentados por los estudiantes, fue posible identificar nuevas percepciones, sentimientos, conocimientos y puntos a mejorar durante la simulación.

*Vinimos y no me imaginaba que se daría una situación así para actuar, en ese momento estaba un poco nervioso, sin saber qué hacer.* (E20)

*Al principio tenía un poco de miedo de que fuera complicado, pero no lo fue.* (E1)

Al mismo tiempo, los estudiantes demuestran satisfacción al participar en la experiencia simulada:

*¡Me encantó! Es muy valioso aprender esto aquí para luego poder aplicarlo en la práctica, puede que incluso nos pongamos un poco nerviosos, pero cuando lleguemos al hospital recordaremos lo que pasó aquí, ‹ahora hago esto, esto y esto›, como aprendí en clase.* (E13)

Además, los participantes discutieron cómo acercar el escenario a la realidad práctica y ayudar a adquirir conocimientos.

*Fue bueno porque estamos más cerca de la realidad, de la práctica, aunque no estemos en el hospital, nos exigen como si estuviéramos allí, entonces terminamos prestando más atención a los detalles, está más cerca de la realidad.* (E26)

*En la simulación, además de tener esta proximidad a la realidad, veo que podemos pensar más allá; Discutimos con otras personas, vemos si piensan o actúan diferente a lo que esperamos o aprendemos con los libros.* (E24)

A partir de este contexto, los participantes pudieron reflexionar sobre los puntos positivos vividos en el escenario, como aspectos relacionados con la resolución del problema presentado, la acogida de los usuarios, la comunicación y el trabajo en equipo, como se muestra a continuación:

*Sentí que mi actuación era apropiada para la situación; Identificamos el problema apenas ingresamos a la habitación y solicitamos una evaluación médica, por lo que, para esa situación específica, era lo correcto.* (E10)

*El tema de acoger al paciente, al acompañante, creo que lo acogemos muy bien.* (E1)

*También pensé que hubo una comunicación rápida con el médico, con los profesionales que en ese momento eran responsables.* (E26)

*Creo que trabajamos bien como equipo, los dos estamos conectados con el médico.* (E2)

Sin embargo, los estudiantes también plantearon puntos negativos/límites que dificultaban la prestación de la asistencia al usuario, y se les animó a expresar si harían algo diferente a lo hecho y cómo podrían mejorar sus acciones. Las intervenciones mostraron aspectos encaminados a mejorar la investigación de los síntomas que relata el usuario, con la realización de un examen físico completo, a fin de garantizar la seguridad en la identificación y presentación personal.

*Se debió descubrir a la paciente para valorar su picor corporal... preguntaron si sentía algo más, pero no lo tomaron en cuenta.* (E23)

*Desde el principio debieron incluir en la ficha de la paciente que era alérgica, ¡siempre debemos resaltar la alergia del paciente!* (E12)

*Ellos también deberían haberse presentado, lo primero que se hace al llegar a la habitación: ‘Soy fulano de tal’.* (E12)

Estos puntos antes mencionados fueron ampliamente discutidos durante el *Debriefing*, asociando la teoría con la práctica experimentada durante la simulación.

### Contribuciones de la simulación a la práctica clínica

En el último momento de la investigación, tras la inmersión de los estudiantes en el campo práctico, los 27 estudiantes fueron invitados a participar en entrevistas individuales, realizadas por la propia investigadora principal. Los estudiantes identificaron que la participación en el escenario simulado, previo a la realización de las inmersiones en el campo de prácticas del área hospitalaria, les brindó una base para sus acciones durante la práctica clínica. De esta manera, se generaron nuevos códigos y temáticas a partir de las entrevistas.

Los estudiantes informaron que pudieron comunicarse mejor y trabajar en equipo, por lo que sugirieron la inclusión de más simulaciones en el plan de estudios, como se presenta en los discursos a continuación.

*Primero, creo que debería haber más simulaciones, porque si tomas al estudiante y lo llevas de la teoría a una simulación en el laboratorio, corregirá sus errores; y luego, al llevarlo a la práctica, creo que tendríamos enfermeros mucho más calificados.* (E11)

*Creo que los profesores deberían hacer más simulaciones en el laboratorio, eso ayudaría mucho al alumno, sería mucho más interesante; los estudiantes aprenderían más y romperían barreras.* (E5)

En seguida, los estudiantes hablaron sobre los beneficios del escenario durante su inmersión en el campo de las prácticas profesionales.

*Todas las fallas y los puntos trabajados durante la simulación… comencé a prestar más atención en eso durante las prácticas, así fue más fácil, aprender el lenguaje corporal, hablar con más calma, mirar a la gente a los ojos, ver si la persona realmente entendía lo que querías decir. Así que mejoré en eso.* (E2)

*Hubo un día durante las prácticas que a mis compañeros se les olvidó presentarse y entonces recordé mucho la simulación, a eso le presté atención.* (E26)

*Lo utilicé en diálisis, mejoré en la comunicación y el cambio de turno, porque a veces hay fallas. Hoy puedo prestar más atención a mis compañeros, se ha vuelto mucho más fácil.* (E5)

*La simulación me ayudó mucho porque antes no trabajábamos juntos, se trataba de un trabajo individual, entonces después de esta práctica profesional nos ayudamos más y la comunicación fue mucho mejor.* (E14)

Finalmente, los estudiantes destacaron sus aportes al aprendizaje como futuros profesionales que se desempeñan en el mercado laboral.

*Me ayudará mucho porque empiezas a tener una perspectiva diferente del trabajo en equipo, de la forma de comunicarte, de hablar con el equipo médico; entonces eso nos termina ayudando en varios puntos, como líder, haciéndonos cargo de la situación y pudiendo liderar con confianza y transmitir esa seguridad al equipo.* (E18)

*Creo que me ayudará mucho porque el trabajo en equipo y la comunicación es lo principal en enfermería, es todo lo que se necesita para llegar al paciente; ¡No hay manera, sin comunicación es imposible! Creo que tendré más confianza para trabajar como enfermero, para realizar mi trabajo.* (E23)

## Discusión

Este estudio aplicó una experiencia simulada con estudiantes de enfermería para desarrollar habilidades de comunicación y trabajo en equipo. Los resultados permitieron identificar dos grandes categorías temáticas: escenario simulado para el desarrollo de la comunicación y el trabajo en equipo en estudiantes de enfermería; y contribuciones de la simulación a la práctica clínica. Los resultados corroboran la implementación de la simulación realista en estudiantes de enfermería como fundamental para la comprensión y el perfeccionamiento de habilidades, comunicación y trabajo en equipo, esenciales para la práctica profesional y colaborativa en el contexto hospitalario.

Se evidenció que los estudiantes lograron desarrollar habilidades desde el primer contacto con el ambiente de simulación: realizaron comunicación y trabajo en equipo con el enfermero del turno nocturno, con la paciente y su acompañante, con el médico y al identificar el cuadro alérgico de la paciente, que requería resolución inmediata. La enfermería requiere una interacción efectiva entre el equipo y el usuario, y la simulación propicia un ambiente controlado y seguro para que los estudiantes experimenten situaciones reales, aprendan a gestionar conflictos, mejoren la asertividad en la comunicación y fortalezcan la cooperación interprofesional^(16)^.

En el *debriefing*, los discursos también revelaron emociones, sentimientos y percepciones del aprendizaje de los estudiantes durante la experiencia simulada, lo que puede ayudar en la discusión de otras simulaciones y/o investigaciones, así como contribuir a la reflexión de los educadores para que tengan la oportunidad de enriquecer o replicar dicha actividad. Estudios afirman que la simulación clínica se ha convertido en uno de los enfoques más utilizados en la formación en enfermería, ya que permite a los estudiantes adquirir mayor conocimiento y confianza para actuar, además de brindar profundidad teórica a través de una escena^(16)^.

Aunque las actividades simuladas contribuyan al perfeccionamiento de habilidades, actitudes y conocimientos, se reconoce que también tienen el potencial de generar miedo, ansiedad e incertidumbre^(17)^, habiéndose identificado cierta ansiedad en los estudiantes que participaron en esta investigación. De este modo, es importante que los estudiantes se familiaricen con la propuesta de simulación, sus objetivos, el entorno simulado y las tecnologías disponibles. Corresponde al docente sensibilizarlos en ese momento para el intercambio de experiencias y la construcción de nuevos conocimientos^(18)^.

Asimismo, la aproximación entre simulación y realidad práctica conlleva la buena receptividad del estudiante a esta metodología^(19)^, ya que puede transportarlo a situaciones que se asemejan a la realidad, en las que se permite cometer errores, repetir procesos, discutir sobre el cuidado al paciente de forma segura y que promueven una formación más sólida integrando teoría y práctica^(20)^. Así, durante la simulación, “aprender sobre, con unos y otros” posibilita una práctica colaborativa y una asistencia eficaz, como recomienda la Educación Interprofesional^(14)^.

Paralelamente, las unidades contextuales también revelaron testimonios, demostrando que los estudiantes reflexionaron sobre qué actitudes podrían haberse llevado a cabo de manera diferente; se demostró la ausencia de algunas acciones importantes que fueron entendidas como limitaciones de su participación en el escenario, tales como: investigar la queja del usuario, realizar un examen físico completo, asegurar la correcta identificación y presentación personal.

Se sabe que el crecimiento de los estudiantes considera aspectos que pueden perfeccionarse, llevando a la identificación de posibles falencias en su aprendizaje. Esto resalta la importancia de la simulación^(21)^, junto a la inclusión de diferentes situaciones y enfoques, incluso en los primeros años de la formación profesional, que pueden incentivar a los estudiantes a discutir mejor las acciones a tomar con el paciente, sus familiares y el equipo profesional. Además, el *feedback* de los docentes es fundamental y permite a los estudiantes reflexionar sobre sus prácticas y comportamientos profesionales^(22)^. De esta manera, el uso de la simulación trajo contribuciones esenciales para el aprendizaje de los estudiantes al fomentar el pensamiento crítico y reflexivo, lo que amplió su conciencia y corresponsabilidad en el perfeccionamiento de las habilidades requeridas en el ejercicio profesional, como la comunicación y el trabajo en equipo.

Por lo tanto, los estudiantes de graduación, al tener la oportunidad de experimentar la simulación, antes de la realización de la práctica profesional, pueden mejorar sus conocimientos y habilidades, reflexionar sobre su rol profesional y aprender a trabajar de manera colaborativa, contribuyendo significativamente a la formación de su identidad profesional^(23)^. Además, estudios^(24-25)^ demuestran que los estudiantes que participaron en actividades simuladas mostraron más conocimientos que aquellos que no lo hicieron. Esto pone de relieve la importancia de llevar a cabo formación/capacitación de forma regular, para garantizar una retención eficaz del conocimiento a largo plazo. Para ello es necesario contar con un cuerpo docente cualificado.

Los investigadores describen que la logística y el financiamiento constituyen el principal obstáculo para la adopción de actividades simuladas en las instituciones educativas^(26)^. Otros sostienen que la introducción de la simulación clínica es un desafío para los educadores, ya que requiere tiempo y esfuerzo para la capacitación docente, como así también para la preparación y definición de los objetivos de aprendizaje y la ejecución de la simulación^(27)^.

Sin embargo, los escenarios simulados están intrínsecamente ligados a la capacidad creativa y al compromiso del docente, tanto en su preparación como en su implementación, por lo que se trata de un valioso recurso didáctico, siempre y cuando se basen en principios teóricos^(28)^. Por lo tanto, es fundamental incluir debates en los proyectos político-pedagógicos de los cursos de Educación Interprofesional y el uso de simulación realista, con el objetivo de preparar adecuadamente a profesores y futuros profesionales de enfermería para la colaboración en los servicios de salud.

El escenario de este estudio presenta un caso/evento en el ambiente hospitalario, lo que puede limitar su uso en otras áreas, como la salud pública, aunque la comunicación y el trabajo en equipo son fundamentales en cualquier lugar de trabajo.

Este estudio contribuye al avance del conocimiento científico, ya que ofrece a las carreras de pregrado en enfermería la oportunidad de explorar y desarrollar la comunicación y el trabajo en equipo en los estudiantes, a través de un escenario validado. Así, podrán ampliar sus conocimientos mediante una actividad realista desarrollada en el laboratorio. Además, la simulación puede ayudar a los estudiantes a desarrollar una visión más integral del rol del enfermero en el entorno hospitalario y guiar a este futuro profesional hacia una práctica segura y colaborativa.

## Conclusión

Con el objetivo de educar y desarrollar/perfeccionar habilidades de comunicación y trabajo en equipo en estudiantes de enfermería para una práctica interprofesional y colaborativa, se empleó un escenario simulado a una paciente hospitalizada en una unidad clínica médica en el cual el estudiante tuvo la oportunidad de aplicar estas habilidades ante un acontecimiento inesperado. De esta manera, la simulación proporcionó mayor comprensión a los estudiantes, al fomentar el análisis crítico y reflexivo de la comunicación y el trabajo en equipo durante la práctica profesional hospitalaria, advirtiéndose la relevancia de su uso incluso en la educación universitaria.
